# Imaging-Based Risk Stratification for Sudden Cardiac Death in Hypertrophic Cardiomyopathy: Current Evidence and Clinical Perspectives

**DOI:** 10.3390/medsci14030390

**Published:** 2026-07-14

**Authors:** Èlia Rifé-Pardo, Sara Sóñora-López, Guillem Casas, Maria Soledad Ceballos, Leandro Santiago Videla, Javier Limeres-Freire, Gisela Teixidó-Tura, Ignacio Ferreira-González, José F. Rodríguez-Palomares

**Affiliations:** 1 Department of Cardiology, Hospital Universitari Vall d’Hebron, 08035 Barcelona, Spain; elia.rife@vallhebron.cat (È.R.-P.); sara.sonora@vallhebron.cat (S.S.-L.); javier.limeres@vallhebron.cat (J.L.-F.); gisela.teixido@vallhebron.cat (G.T.-T.); ignacio.ferreira@vallhebron.cat (I.F.-G.); 2 Cardiovascular Imaging Unit and Inherited Cardiovascular Diseases Unit, Cardiology Department, Vall d’Hebron Institut de Recerca (VHIR), Hospital Universitari Vall d’Hebron, 08035 Barcelona, Spain; mariasoledadceballosp@gmail.com (M.S.C.); leandro_santiago_videla@hotmail.com (L.S.V.); 3 Biomedical Research Networking Centre on Cardiovascular Diseases (CIBERCV), Instituto de Salud Carlos III, 28029 Madrid, Spain; 4 Cardiovascular Diseases, Vall d’Hebron Institut de Recerca (VHIR), Hospital Universitari Vall d’Hebron, 08035 Barcelona, Spain; 5 Department of Medicine, Universitat Autònoma de Barcelona, 08023 Barcelona, Spain; 6 Epidemiology and Public Health Networking Biomedical Research Centre (CIBERESP), Instituto de Salud Carlos III, 28029 Madrid, Spain

**Keywords:** hypertrophic cardiomyopathy, sudden cardiac death, imaging, late gadolinium enhancement, strain

## Abstract

Hypertrophic cardiomyopathy (HCM) is a common inherited myocardial disorder and a leading cause of sudden cardiac death (SCD) across all age groups. Accurate risk stratification remains a clinical challenge, as conventional algorithms fail to fully capture the heterogeneous arrhythmic substrate of the disease. In recent years, cardiac imaging has emerged as a pivotal tool for refining SCD prediction by providing detailed structural, functional, and tissue-characterisation data. This review offers a comprehensive synthesis of imaging-derived predictors of SCD in HCM. We examine established echocardiographic markers, including maximal left ventricular wall thickness, left ventricular ejection fraction and left ventricular outflow tract obstruction, together with advanced cardiovascular magnetic resonance parameters such as late gadolinium enhancement and apical aneurysm detection. In addition, we discuss the emerging role of extracellular volume expansion, quantitative mapping techniques, and strain abnormalities within the framework of contemporary risk stratification models. By bridging imaging biomarkers with underlying pathophysiological mechanisms, this review highlights current evidence, limitations, and future directions to improve precision in SCD risk assessment in HCM.

## 1. Introduction

Hypertrophic cardiomyopathy (HCM) is a cardiac disease with a high prevalence (1:500 in adults) and pronounced genetic and phenotypic heterogeneity [1,2,3]. In adults, it is characterised by unexplained left ventricular (LV) wall hypertrophy (maximum myocardial wall thickness [MWT] of ≥15 mm, or ≥13 mm in the presence of family history or electrocardiogram abnormalities) [1,2]; however, recent evidence suggests that demographic-based personalized LV hypertrophy (LVH) thresholds may improve diagnostic accuracy. Adjusting for age, sex, and body surface area may provide more discriminative values [4]. In children, the diagnostic criteria must be adjusted for body size and growth. According to the European guidelines, an MWT greater than two standard deviations (SD) above the predicted mean (z-score > 2) is considered diagnostic [1]. In contrast, the American guidelines recommend a z-score > 2 for children with a definitive family history of HCM and a threshold of >2.5 for those without such a history [2]. In approximately half of cases, pathogenic variants in cardiac sarcomeric genes can be identified, exhibiting autosomal-dominant Mendelian inheritance with variable penetrance [5,6].

Adult patients with HCM exhibit excess mortality compared with the general population, which is more pronounced in females [7]. Sudden cardiac death (SCD), with an annual incidence of approximately 0.4–0.5%, represents the principal cause of mortality in younger patients [8,9]. In contrast, heart failure (HF)-related death occurs throughout life and becomes increasingly prevalent in older individuals [7,9].

Currently, the decision to implant an implantable cardioverter-defibrillator (ICD) for primary prevention is primarily based on risk stratification using either the European Society of Cardiology (ESC) 5-year SCD risk calculator or the American College of Cardiology/American Heart Association (ACC/AHA) algorithm [1,2]. According to the ESC model, patients are classified into three risk groups: low risk (<4% risk of SCD over 5 years, where ICD is not recommended in the absence of additional risk factors such as left ventricular ejection fraction (LVEF) below 50% or extensive late gadolinium enhancement (LGE), defined as >15% of total LV mass), intermediate risk (4–6% risk of SCD over 5 years, ICD may be considered) and high risk (>6% risk of SCD over 5 years, ICD should be considered) [1]. Conversely, according to the ACC/AHA, the presence of any risk factor, including family history of HCM-related SCD, highly suspicious arrhythmic syncope, an LV apical aneurysm, LV dysfunction, massive LVH, evidence of non-sustained ventricular tachycardia (NSVT), or extensive LGE, warrants consideration for ICD implantation [2]. In any case, both models are imperfect. The ACC/AHA strategy has shown greater sensitivity, whereas the ESC model appears more specific, thereby reducing unnecessary implantations in low-risk patients [10].

Despite substantial advances in understanding SCD, its genetic substrate, and associated risk markers, current risk-stratification strategies remain imperfect. In this review, we focus on cardiac imaging markers related to SCD and critically appraise the underlying evidence, aiming to provide a comprehensive framework to improve individualized risk assessment and refine primary prevention ICD decision-making (Central Illustration, Table 1).

## 2. Materials and Methods

For this narrative review, relevant literature was identified through a structured search of the PubMed database using the terms “hypertrophic cardiomyopathy”, “cardiac imaging markers”, and “sudden cardiac death”. No date restrictions were applied to the search. A total of 250 articles were screened, and original studies and meta-analyses evaluating cardiac imaging markers and their association with SCD in HCM were considered for inclusion. Paediatric studies and case reports were excluded. Articles addressing echocardiographic and cardiovascular magnetic resonance parameters with prognostic implications were selected based on their relevance and scientific robustness. The schematic illustrations included in this manuscript were created using BioRender Premium (BioRender, Toronto, ON, Canada). All other clinical images were obtained from our institutional Cardiovascular Imaging Unit within the Department of Cardiology.

## 3. Maximum Myocardial Wall Thickness

As previously discussed, unexplained asymmetric LVH constitutes the diagnostic hallmark of HCM. The most common location of hypertrophy is the confluence of the basal anterior septum with the contiguous anterior free wall, followed by the posterior septum at the midventricular level [71]. Some patients exhibit diffuse hypertrophy involving more than 50% of the LV myocardium, while a minority may demonstrate focal increased wall thickness without an associated increase in overall LV mass [71]. Over one-third of HCM patients also exhibit increased right ventricular (RV) wall thickness [72], most commonly at the RV insertion points into the anterior or posterior septum. However, the entire RV may be involved in some cases, and diffuse RV involvement may also occur. MWT is one of the variables included in the ESC risk calculator for SCD [1,73,74] and in the ACC/AHA guidelines algorithm [2]. According to the latter, in the presence of massive LVH (maximum MWT ≥ 30 mm in any segment), a primary-prevention ICD should be considered (Class IIa recommendation) [2] (Table 2).

**Figure 1:**
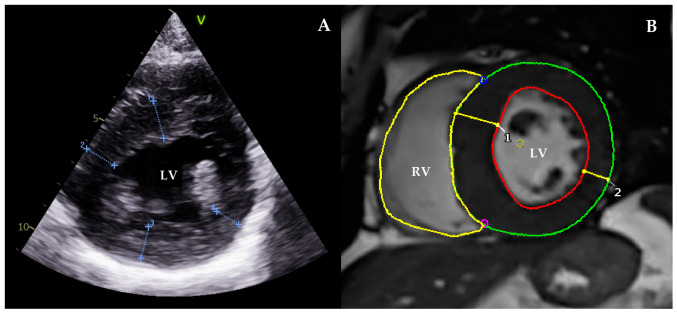
Short-axis maximal MWT measurement by TTE (**A**) and CMR (**B**). In the TTE image, 1 and 2 indicate the interventricular septum and 3 and 4 indicate the posterior wall. In the CMR image, 1 indicates the interventricular septum and 2 indicates the posterior wall. CMR: Cardiac magnetic resonance; LV: Left ventricle; RV: Right ventricle; MWT: Myocardial wall thickness; TTE: Transthoracic echocardiography.

Transthoracic echocardiography (TTE) is the first-line imaging technique and is recommended in all patients with suspected HCM (Class I recommendation) [1,2]. Cardiovascular magnetic resonance (CMR) imaging offers superior anatomic resolution and is also recommended at initial presentation (Class I recommendation) [1,2]. However, TTE and CMR have been shown to exhibit significant discrepancies in MWT assessment, with CMR currently considered the gold standard [75,76,77]. Bois et al. have reported identical MWT measurements with both techniques in only 12% of cases, with a non-trivial median difference of 3 mm between the two modalities [75]. MWT should be measured and confirmed in two different planes (short- and long-axis, Figure 1).

Early studies demonstrated a marked increase in SCD risk with extreme LVH [11,12,13], reporting near-zero risk in patients with wall thickness ≤15–19 mm and cumulative rates approaching 40% at 20 years in those with ≥30 mm [11]. This cut-off was reproduced in subsequent studies and associated with hazard ratios (HRs) ranging from approximately 3.0 to 3.5, particularly in younger patients [13,14], supporting its early use as a criterion for primary-prevention ICD implantation.

However, later evidence challenged this dichotomous approach. Several studies failed to confirm a significant independent association between extreme LVH and SCD [15,16,17,18], and others demonstrated that most SCD events occurred in patients with MWT < 30 mm [12]. These findings suggested that wall thickness should be considered a continuous rather than a binary risk marker [12,19]. Indeed, although SCD risk increases progressively with greater MWT, the incremental risk per 5 mm increase is modest compared with the impact of clinical risk factors (relative risk per additional risk factor of 2.0 vs. 1.3 per 5 mm increase in MWT) [19]. Patients with MWT ≥30 mm but no additional risk factors have a relatively low absolute risk (5%), whereas those with multiple clinical risk factors experience substantially higher event rates regardless of wall thickness (34% when MWT ≥ 30 mm and three additional risk factors) [19].

Consequently, MWT alone (particularly when defined by an arbitrary cut-off) appears insufficient for risk stratification, and its prognostic value is better interpreted within a multifactorial, continuous risk framework. This conceptual shift explains why extreme LVH was not retained as an independent indication for prophylactic ICD implantation in subsequent clinical guidelines [1,78].

The mechanisms by which myocardial hypertrophy may contribute to or cause SCD are not fully understood. Nevertheless, it can be expected that the greater the hypertrophy, the more significant the disruption of myocardial architecture and the increase in myocardial oxygen demand. A helical distribution of hypertrophy has been associated with left ventricular outflow tract obstruction (LVOTO) and systolic anterior motion (SAM) of the mitral valve, both of which are significantly associated with NSVT [79].

On the other hand, the traditional diagnostic criteria exhibit low sensitivity for apical HCM, as it often presents with less pronounced LVH. More accurate criteria have been proposed by Hughes et al., using per-segmental apical wall thickness [80]. The ESC risk score has not been validated for apical HCM. Compared with non-apical HCM, the apical variant carries a more favourable prognosis [81,82,83].

Septal reduction therapies (SRTs), including surgical myectomy and alcohol septal ablation (ASA), reduce LV wall thickness and LVOTO and may therefore modify established SCD risk profiles. Long-term SCD rates after SRT are low, estimated at approximately 0.4% per year after ASA and 0.5% per year after myectomy [84]. Data on ASA is conflicting, with reports suggesting either a protective effect [85] or an increased arrhythmic burden related to the iatrogenic infarction [86]. In patients undergoing ASA, Liebregts et al. validated the HCM Risk-SCD model, showing good agreement between predicted and observed 5-year SCD risk (5.1% vs. 4.0%) and identifying pre-procedural maximal wall thickness ≥30 mm as an independent predictor of SCD (HR 3.48) [87]. In surgical myectomy cohorts, evidence is inconsistent. McLeod et al. reported a marked reduction in SCD incidence compared with non-myectomy patients (0.24% vs. 4.3% per year), likely reflecting improved haemodynamics and regression of key risk markers such as wall thickness and left ventricular outflow tract (LVOT) gradient [88,89,90,91]. However, another study showed no significant benefit [92]. Overall, arrhythmic risk appears to be driven more by the patient’s baseline risk profile than by the obstruction-relief procedure itself [93].

Cardiac myosin inhibitors (CMIs) are emerging novel therapies, with early evidence suggesting benefits in LV mass reduction and obstruction relief; however, their impact on current risk stratification paradigms remains uncertain and warrants further study. In a CMR substudy of EXPLORER-HCM [94], Saberi et al. demonstrated that mavacamten reduced LV mass index by −17.4 g/m^2^ and MWT by −2.4 mm compared with placebo [95]. Similarly, the VALOR-HCM trial showed sustained reductions in LV mass index of approximately −13 g/m^2^ at 32 weeks [96]. These changes were sustained when evaluated at 56 [97] and 128 weeks [98]. Trials with aficamten have also reported significant decreases in MWT, alongside reductions in LV mass index [99,100,101,102,103].

Taken together, these data indicate that both surgical and pharmacological therapies can shift patients along the hypertrophy continuum. As wall thickness and myocardial mass are no longer static parameters, current SCD risk models (derived mainly from untreated populations) may need to be recalibrated in future studies to account for therapy-induced myocardial remodelling.

## 4. Left Ventricular Outflow Tract Obstruction

**Figure 2:**
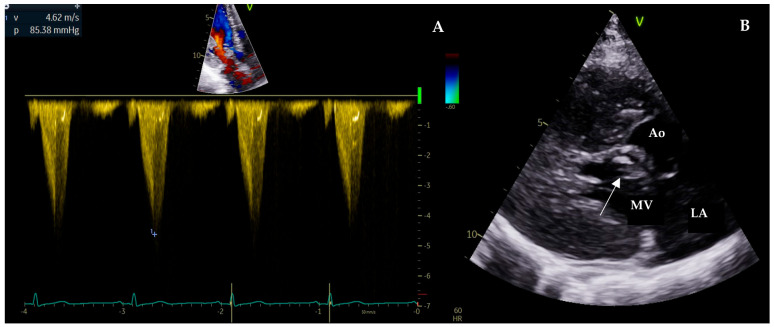
(**A**) LVOT dynamic gradient (85 mmHg, Vmax 4.62 m/s) measurement with the continuous doppler by TTE (number 1). (**B**) Presence of SAM of the mitral valve (arrow) assessed by TTE. Ao: Aorta; LA: Left atrium; LVOT: Left ventricle outflow tract; MV: Mitral valve; SAM: Systolic anterior movement; TTE: Transthoracic echocardiography; V: Velocity.

HCM is classified as obstructive or non-obstructive based on the presence of significant LVOTO, defined as a peak gradient ≥ 30 mmHg at rest or with provocation [1,2], while a gradient ≥ 50 mmHg warrants specific treatment (Figure 2). Even though both entities have been associated with arrhythmic events and SCD, LVOTO is included as a predictor of adverse events in clinical guidelines and major arrhythmic risk calculators [1,2,73]. This is supported by many studies linking midventricular or LVOTO to higher arrhythmic risk and overall SCD [18,20,21], and in particular to exercise-unrelated SCD [16]. In a cohort of 917 HCM patients (288 with LVOTO), Elliott et al. reported that LVOTO was associated with a 2.4-fold higher risk of SCD or appropriate ICD therapy [21]. Nevertheless, the evidence remains inconsistent, with some studies failing to confirm LVOTO as an independent risk factor after adjusting for other well-established predictors, such as syncope, marked MWT, or a modest rise in blood pressure during exertion [11,15,104,105]. Indeed, owing to this inconsistency, the ACC/AHA guidelines emphasize that obstruction should be considered primarily as a determinant of symptoms and as an indication for specific treatment and/or SRT, rather than as an independent significant risk factor for SCD [2] (Table 2). What appears clearer is the association between LVOTO and progression to end-stage HF [11,15,18,22,23], as well as new-onset atrial fibrillation (AF) [24].

As stated before, whether different SRTs for relieving LVOTO influence the risk of SCD and arrhythmic events remains unresolved, with contradictory data [84,85,86,87,88,89,90,91,92,93].

Regarding pharmacological therapy, management of LVOTO is primarily based on beta-blockers, with nondihydropyridine calcium channel blockers as alternatives [1]. While these agents effectively reduce LVOT gradients and improve symptoms, their impact on SCD prevention remains uncertain. A meta-analysis of 21 studies involving 775 patients showed that beta-blockers significantly reduced LVOT gradients and improved functional status but did not consistently reduce mortality [106]. Consequently, current guidelines recommend beta-blockers mainly for symptomatic ventricular arrhythmias or after appropriate ICD therapies, rather than as a strategy for primary SCD prevention [107].

Along the same lines, the novel CMIs have demonstrated rapid and intense reductions in LVOTO [94,97]. Still, larger, longer studies are needed to determine whether these hemodynamic effects correlate with a decrease in arrhythmic burden.

## 5. Apical Aneurysms

**Figure 3:**
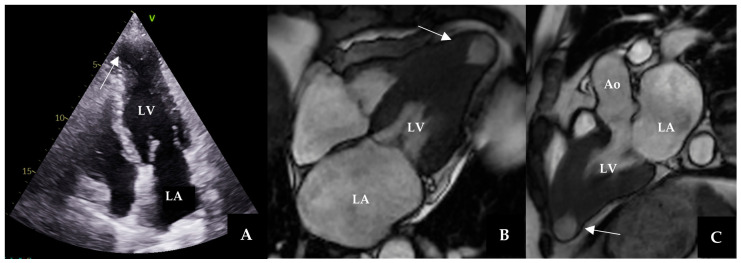
Apical aneurysm (arrow) in a 2−chamber view by TTE (**A**) and in 4−chamber (**B**) and 3−chamber (**C**) views by CMR. Ao: Aorta; CMR: Cardiac magnetic resonance; LA: Left atrium; LV: Left ventricle; TTE: Transthoracic echocardiography.

LV apical aneurysms are rare complications that occur in 2–5% of HCM patients [25,26], with CMR representing the gold standard for their detection (Figure 3). The development of apical aneurysms has been linked to midventricular obstruction, elevated LV mass, increased wall thickness, progression of LGE extent, and microvascular apical ischaemia [27].

Apical aneurysms have been associated with clinical adverse events, including SCD, ventricular arrhythmias, appropriate ICD discharge, thromboembolic stroke, progressive HF, and death [25,26,27,28,29,30,31]. In a meta-analysis including 2382 patients with HCM, 2% of whom had an apical aneurysm, they were found to be associated with a significantly increased risk of arrhythmic and SCD-related events (OR 4.67) [28]. Some studies suggest that the underlying substrate for arrhythmogenesis arises from the aneurysm’s scarred border, in conjunction with extensive myocardial fibrosis [25]. Larger apical aneurysm size has been linked to higher arrhythmic risk [29,31,32], although no definitive cut-off has been established [26]. Sherrid et al. identified >4 cm^2^ as a high-risk threshold; in their cohort, patients with aneurysms > 4 cm^2^ had markedly higher 5-year arrhythmic event rates than those with ≤2 cm^2^ (35% vs. 6%) [32].

The ACC/AHA guidelines for HCM include apical aneurysms in their SCD risk algorithm and as an independent risk factor [2]. In contrast, the ESC guidelines emphasize that the presence of an apical aneurysm alone is insufficient to justify ICD implantation, citing evidence derived mainly from small, retrospective cohorts with potential selection bias and the frequent coexistence of additional risk markers [1,26,29] (Table 2). Reflecting this uncertainty, Rowin et al. reported that among 21 patients with apical aneurysms who experienced arrhythmic events, 13 (62%) were classified as low risk by the HCM Risk-SCD model (<4% estimated 5-year risk) and therefore would not have met ESC criteria for prophylactic ICD implantation [26].

## 6. Left Atrium

Left atrium (LA) enlargement is observed in approximately one-third of patients with HCM [33]. The underlying causes of LA changes in function and structure are multifactorial; however, the predominant contributing mechanisms are SAM of the mitral valve, often resulting in mitral regurgitation (MR), and diastolic dysfunction with elevated filling pressures [108]. LA remodelling is characterised by increased LA volume, reduced atrial strain, and prolonged conduction times [109], leading to a recognized “atrial myopathy”.

**Figure 4:**
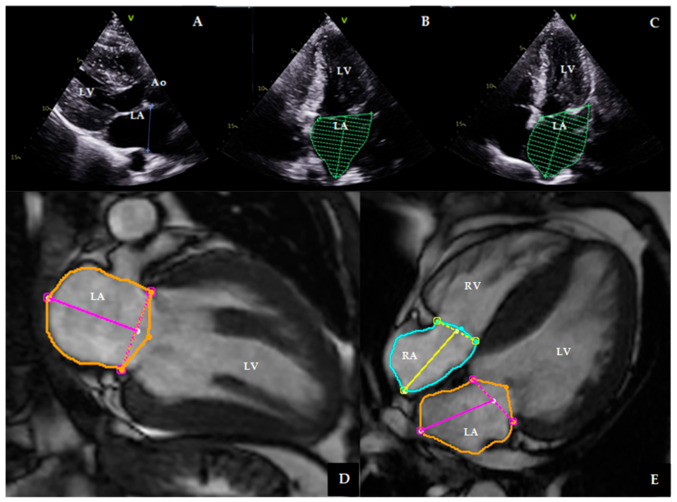
(**A**) LA anteroposterior diameter measurement by TTE. (**B**) Two−chamber and (**C**) 4−chamber LA volume measurement by TTE. (**D**) Two−chamber and (**E**) 4−chamber LA and RA volume measurement by CMR. The orange lines represent the LA perimeter, the pink lines represent the maximal LA diameter, the blue line represents the RA perimeter, and the yellow line represents the maximal RA diameter. Ao: Aorta; CMR: Cardiac magnetic resonance; LA: Left atrium; LV: Left ventricle; RA: Right atrium; RV: Right ventricle; TTE: Transthoracic echocardiography.

LA diameter is a readily obtainable measurement by TTE in most patients. It should be measured at the end-systole and on the parasternal long-axis (Figure 4A) [110,111]. However, although a linear relationship between LA diameter and volume has been described, volume is now considered a more reliable parameter for accurately assessing LA size in HCM patients (Figure 4B,E) [33,108].

The association between LA enlargement and SCD risk has been examined in several studies, yielding mixed results. Increased LA size has been significantly associated with a higher risk of SCD in just a single study [34]. In a multivariate Cox proportional hazards analysis that included unexplained syncope, age, and LV maximal MWT, Spirito et al. identified LA dimension, as a continuous variable, as a statistically significant predictor of SCD, with an HR of 1.03 [34]. Minami et al. found a substantial association between LA enlargement and SCD, observed only in patients without AF, with an adjusted HR of 5.23 [35]. Accordingly, LA size has been recognized as a risk factor in the ESC SCD risk calculator; [1] however, it is not accounted for in the American risk assessment algorithm [2] (Table 2).

Additionally, larger LA sizes, as assessed by both diameter and volume, have been associated with poorer outcomes. Patients with LA enlargement are at increased risk of suffering major adverse cardiovascular events (MACEs) [33,36,37,38] and more frequently present with advanced New York Heart Association (NYHA) functional class, AF, and LVOTO [36].

AF is the most common sustained supraventricular arrhythmia in patients with HCM (annual incidence of 2–4%) [39]. Recently, a specific score (HCM-AF Score) has been developed to stratify the risk of developing AF in this population [112]. Furthermore, chronic AF, as opposed to paroxysmal or persistent forms, has also been linked with an increased risk of SCD (HR of 4.90) [40]. Other larger studies failed to identify a significant relationship between AF and SCD [15,16,18,35].

**Figure 5:**
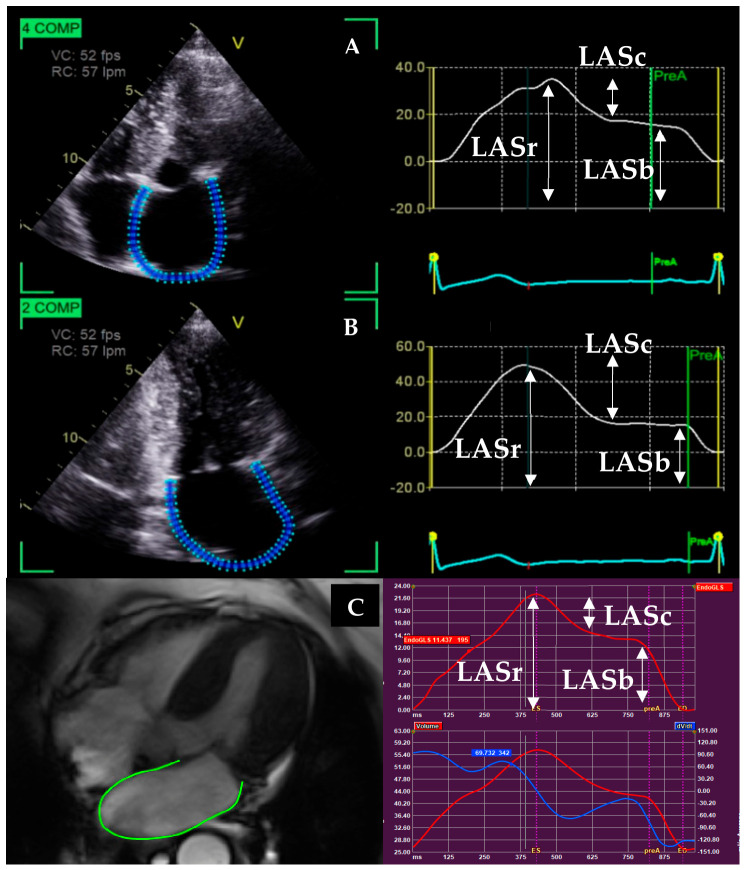
LA strain measurement by TTE and CRM. (**A**) Four−chamber and (**B**) 2−chamber TTE views and their respective R−wave deformation graphics. LA strain analysis is performed using ECG R−wave gating (onset of ventricular systole); with an initial upward deflection reflecting LA filling and stretching during LV systole, followed by a downward slope corresponding to LA emptying. The LA strain curve (white curves in Figure 5A,B and red curves in Figure 5C) comprises three functional phases: the reservoir phase (LASr), during which the LA fills from the pulmonary veins while the mitral valve remains closed; the conduit phase (LASc), in which mitral valve opens and blood passively flows into LV; and the booster phase (LASb), which reflects active atrial contraction. (**C**) Four-chamber CMR view with subsequent strain analysis graphic. The green line represents the perimeter of the LA and the blue line shows the Doppler index (dV/dt) which represents the rate of change in blood flow velocity over time, providing an index of flow acceleration and haemodynamic performance throughout the cardiac cycle. Positive deflections correspond to flow acceleration, whereas negative deflections reflect flow deceleration. CMR: Cardiac magnetic resonance; ECG: Electrocardiogram; LA: Left atrium; LASb: Left atrial longitudinal strain booster; LASc: Left atrial longitudinal strain conduit; LASr: Left atrial longitudinal strain reservoir; LV: Left ventricle; TTE: Transthoracic echocardiography.

Reduced left atrial longitudinal strain (LAS) in HCM is associated with a higher risk of MACE [113,114,115], with LA booster strain showing the strongest prognostic value (Figure 5) [114]. In otherwise low-risk patients, a CMR-derived LA global longitudinal strain > 28.2% has been linked to a favourable prognosis [115]. Impaired LAS also correlates with advanced atrial fibrosis [116] and higher SCD risk: all LAS components (reservoir, conduit, and booster) are significantly reduced in high-risk patients, with reservoir strain emerging as an independent predictor of high SCD risk [117].

Following SRT, early studies reported no significant changes in LA size; [118] however, more recent data demonstrate significant improvements in LA diameter, volume, and strain after surgical myectomy or ASA, supporting a potential role for LA reverse remodelling in risk modification [119,120,121,122,123,124,125].

CMIs have been shown to improve LA dimensions [95,96,98,126,127,128,129]. In the VALOR-HCM trial, continuous mavacamten therapy reduced the LA volume index (LAVI) by −6.8 mL/m^2^, with most of the effect occurring within the first 16 weeks; patients crossed over from placebo showed a similar reduction (−5.5 mL/m^2^) [96,129]. These improvements were sustained up to 128 weeks [97,98]. Consistently, a CMR substudy of EXPLORER-HCM [94] demonstrated a greater reduction in LAVI with mavacamten than placebo (−10.3 mL/m^2^ at 30 weeks) [95]. Comparable reductions in LAVI have also been reported with aficamten [99,101,102]. In contrast, small studies have reported deterioration in LA strain without significant changes in LAVI, possibly related to the direct negative inotropic effects of CMIs on the atrial myocardium [130,131].

Overall, whether treatment-induced changes in LA size and function translate into meaningful modifications of SCD risk stratification remains to be determined.

## 7. Left Ventricular Ejection Fraction

**Figure 6:**
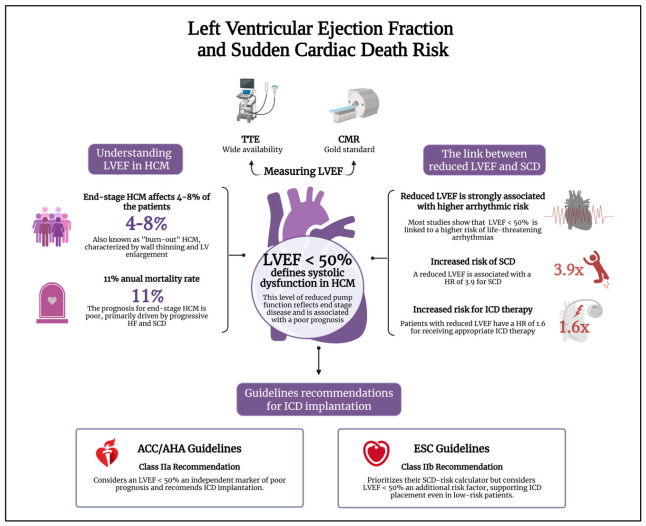
Prognostic impact of impaired LVEF regarding SCD. ACC/AHA: American College of Cardiology/American Heart Association; CMR: Cardiac magnetic resonance; ESC: European Society of Cardiology; HCM: Hypertrophic cardiomyopathy; HF: Heart failure; HR: Hazard ratio; ICD: Implantable cardioverter-defibrillator; LV: Left ventricle; LVEF: Left ventricular ejection fraction; SCD: Sudden cardiac death; TTE: Transthoracic echocardiography.

CMR is considered the gold standard for LVEF measurement; however, TTE is more commonly used because it is readily available [132]. In HCM, LV systolic dysfunction is defined by an LVEF < 50% and typically reflects end-stage disease, which is associated with a poor prognosis (Figure 6).

End-stage or “burned-out” HCM occurs in approximately 4–8% of patients [41,42]. Although the predictors are not fully established, evidence suggests that wall thinning, LV enlargement, and the presence of a pathogenic or likely pathogenic sarcomeric variant increase the risk of progression to this advanced stage [41,43]. Its poor prognosis is primarily driven by progressive HF and SCD, with an annual mortality rate of 11% [42]. In a multicentre study of 6793 patients with HCM (8% of whom had an LVEF < 50%), Marchand et al. reported that, over 8.4 years after the onset of systolic dysfunction, 35% experienced either death or the need for advanced HF therapies [41]. Focusing on arrhythmic events, most published series support an association between reduced LVEF and increased arrhythmic risk. However, the majority have not assessed whether this relationship is independent of other covariables. Some authors have reported appropriate ICD therapies in about 10% of patients listed for heart transplantation [42] and SCD in up to 47% of end-stage HCM patients over a mean follow-up of 5 ± 3 years [133]. Marstrand et al. reported that reduced LVEF was associated with a higher risk of ICD therapy (HR 1.6) and SCD (HR 3.9) [41]. In contrast, Rowin et al. found no significant difference in lethal tachyarrhythmia rates between patients with LVEF 35–49% and those with LVEF < 35% (17% vs. 19%) [134].

The ACC/AHA guidelines consider an LVEF < 50% an independent marker of poor prognosis and recommend ICD implantation as a Class IIa indication [2]. In contrast, the ESC guidelines prioritize the use of the SCD-risk calculator. However, they consider an LVEF < 50% as an additional risk factor, even in low-risk patients (<4%), and support a class IIb recommendation for ICD placement [1] (Figure 6, Table 2).

## 8. Diastolic Function

TTE is the primary imaging technique for assessing diastolic function and LV filling pressures [135,136,137].

However, in HCM, conventional diastolic indices (e.g., transmitral parameters) alone show limited correlation with invasive filling pressures. Alternative measures, such as the difference between pulmonary flow-derived atrial reverse (Ar) and transmitral atrial (A) velocity duration (Ar−A duration) greater than 30 ms, may provide a more accurate prediction of filling pressures [135]. The mitral E/e′ ratio is one of the most widely studied echocardiographic parameters for assessing diastolic function and has a better correlation with filling pressures in HCM.

**Figure 7:**
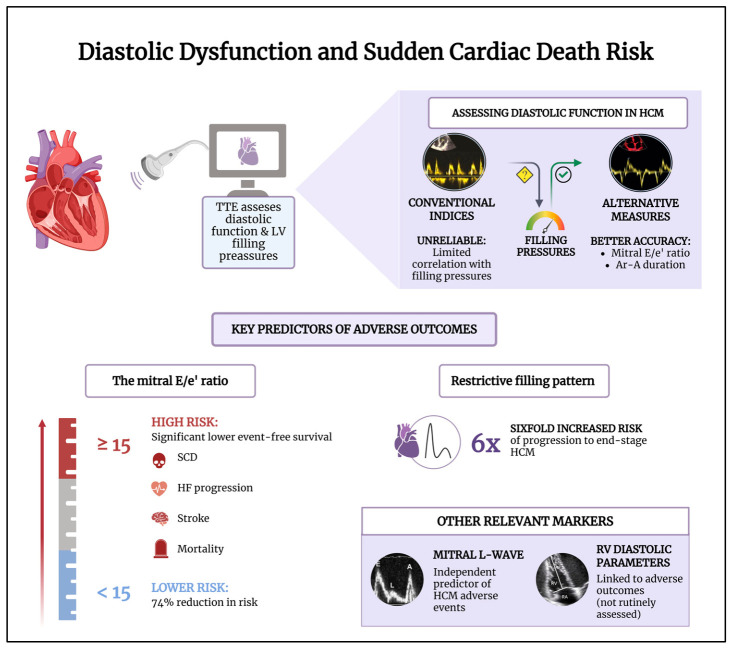
Prognostic impact of impaired diastolic function regarding sudden cardiac death. HCM: Hypertrophic cardiomyopathy; HF: Heart failure; LV: Left ventricle; RV: Right ventricle; SCD: Sudden cardiac death; TTE: Transthoracic echocardiography.

Diastolic dysfunction is consistently associated with worse outcomes in HCM (Figure 7) [44]. An elevated E/e′ ratio has been identified as an independent predictor of MACE, including SCD, HF progression, stroke, AF, and HCM-related mortality [45,46,47,48]. Patients with E/e′ ≥ 15 exhibited significantly lower event-free survival compared with those with E/e′ < 15, corresponding to a 74% higher risk of adverse events; [45] this association has also been observed in paediatric populations [49]. In addition, a restrictive filling pattern is associated with a sixfold increased risk of progression to end-stage HCM, underscoring the prognostic importance of diastolic dysfunction [48].

Although other diastolic parameters have been linked to adverse outcomes, current evidence remains limited. The presence of a mitral L-wave has been reported as an independent predictor of HCM-related events after adjustment for conventional SCD risk markers [138]. Likewise, RV diastolic parameters have been associated with adverse outcomes, including a tricuspid inflow E/E′ ratio > 6.88 and an E-wave deceleration time (DT) < 239 ms; [139] however, these measures are not routinely assessed in clinical practice.

## 9. Strain

**Figure 8:**
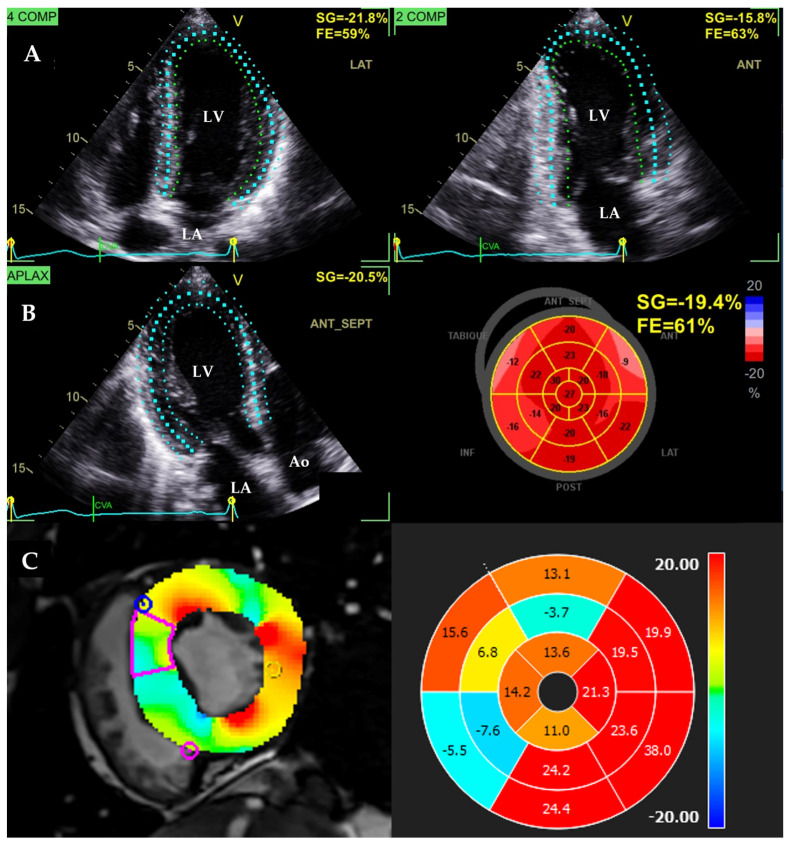
(**A**,**B**) LV longitudinal strain (−19.4%) measurement by TTE. (**C**) LV radial strain measurement using the CMR CVI42 software (Circle Cardiovascular Imaging Inc., Calgary, AB, Canada). Ao: Aorta; CMR: Cardiac magnetic resonance; FE: Fraction ejection; LA: Left atrium; LV: Left ventricle. SG: Strain global; TTE: Transthoracic echocardiography.

Left ventricular global longitudinal strain (LV GLS) is the most consistently reported deformation parameter associated with adverse outcomes (Figure 8). With cut-off values ranging from −10.5% to −15%, impaired LV GLS independently predicts HF hospitalization, LVEF deterioration, transplantation, and mortality [38,50,51,52]. LV GLS > −15% has also been independently associated with SCD and appropriate ICD therapies [51,53]. Additionally, LV GLS correlates with myocardial fibrosis on CMR [54] and with NSVT on Holter monitoring [55]. Regional strain analysis, particularly transmural longitudinal strain in hypertrophic segments, may better predict SCD and ICD shocks than GLS [54]. In contrast, the prognostic value of global circumferential (GCS) and radial strain (GRS) remains inconsistent [140,141]. Despite increasing clinical use, the lack of standardization and universal reference values, including vendor-dependent variability, currently limits the incorporation of echocardiographic strain into guidelines, and LV GLS is therefore considered an emerging marker of SCD risk [111].

**Figure 9:**
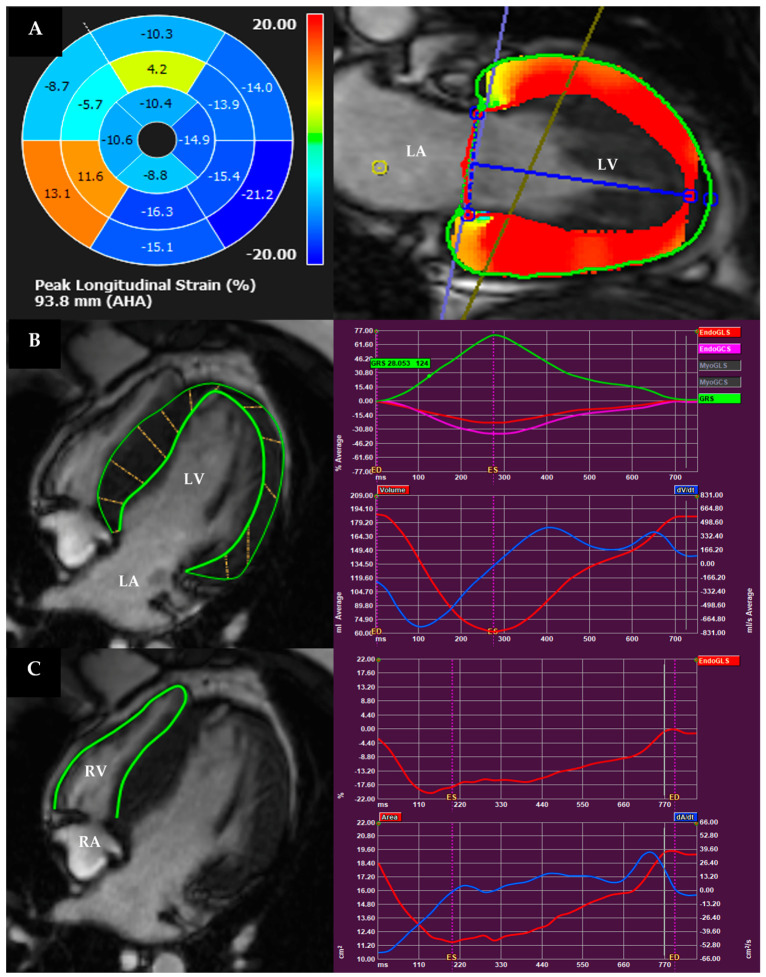
(**A**) LV longitudinal strain measurement in 2−chamber view and strain quantification using the CMR CVI42 software. The colours used in the CMR images are intended for illustrative purposes only and do not represent quantitative measurements, with the exception of the polar map, where the displayed scale accurately quantifies LV longitudinal strain values. (**B**) LV and (**C**) RV longitudinal strain measurements using the CMR MEDIS Suite MR software (MEDIS Medical Imaging Systems, Leiden, The Netherlands). The red lines represent the GLS measurements; the blue lines indicate the Doppler index (dV/dt); the pink line corresponds to GCS; and the green line to GRS. AHA: American Heart Association (17-segment model); CMR: Cardiac magnetic resonance; GCS: Global circumferential strain; GLS: Global longitudinal strain; GRS: Global radial strain; LA: Left atrium; LV: Left ventricle; RA: Right atrium; RV: Right ventricle.

CMR feature tracking (CMR-FT) enables reproducible, chamber-wide strain assessment independent of acoustic windows and is emerging as a promising tool for risk stratification (Figure 8 and Figure 9). LV GLS derived from CMR-FT independently predicts MACE [141,142], including SCD and ventricular arrhythmias [141], even in patients classified as low risk by established clinical models [143].

LAS, particularly the reservoir (LARS) and conduit (LACS) phases, is frequently impaired in HCM and has been associated with MACE and SCD [113,114,115,116,117,144,145,146,147,148]. In some studies, LAS has demonstrated greater sensitivity than LV GLS for non-SCD outcomes [146]. However, evidence specifically addressing arrhythmic risk remains limited. Data on RV strain are scarce, though RV GLS and GCS have been linked to adverse outcomes in selected populations [149]. Diastolic strain assessment is still exploratory, but longitudinal peak diastolic strain rate has shown independent prognostic value for MACE, including SCD [150].

## 10. Late Gadolinium Enhancement

CMR is an essential tool for SCD risk assessment in HCM, as it enables LGE detection of myocardial fibrosis. Although the exact mechanism is unclear, evidence suggests that LGE reflects replacement fibrosis from myocyte loss caused by recurrent microvascular ischemia due to abnormal intramural coronary arteries [6]. Fibrosis in HCM is heterogeneous and includes replacement, interstitial, and perivascular patterns [151]. Importantly, not all LGE necessarily reflects true replacement fibrosis, as extracellular matrix expansion and myocyte disarray may also permit gadolinium accumulation [6,152].

Myocardial fibrosis and the surrounding grey zone are believed to serve as arrhythmogenic substrates that facilitate ventricular arrhythmias and may lead to SCD [56,153,154,155]. LGE is strongly associated with an increased burden of ventricular arrhythmias, including NSVT [156], ventricular ectopy [57,58,59,156,157], and inducible ventricular tachycardia (VT), particularly when a greater extent of LV mass is affected [158]. Given the established link between NSVT and elevated SCD risk [13], the higher arrhythmic burden observed in patients with LGE suggests an increased susceptibility to SCD.

**Figure 10:**
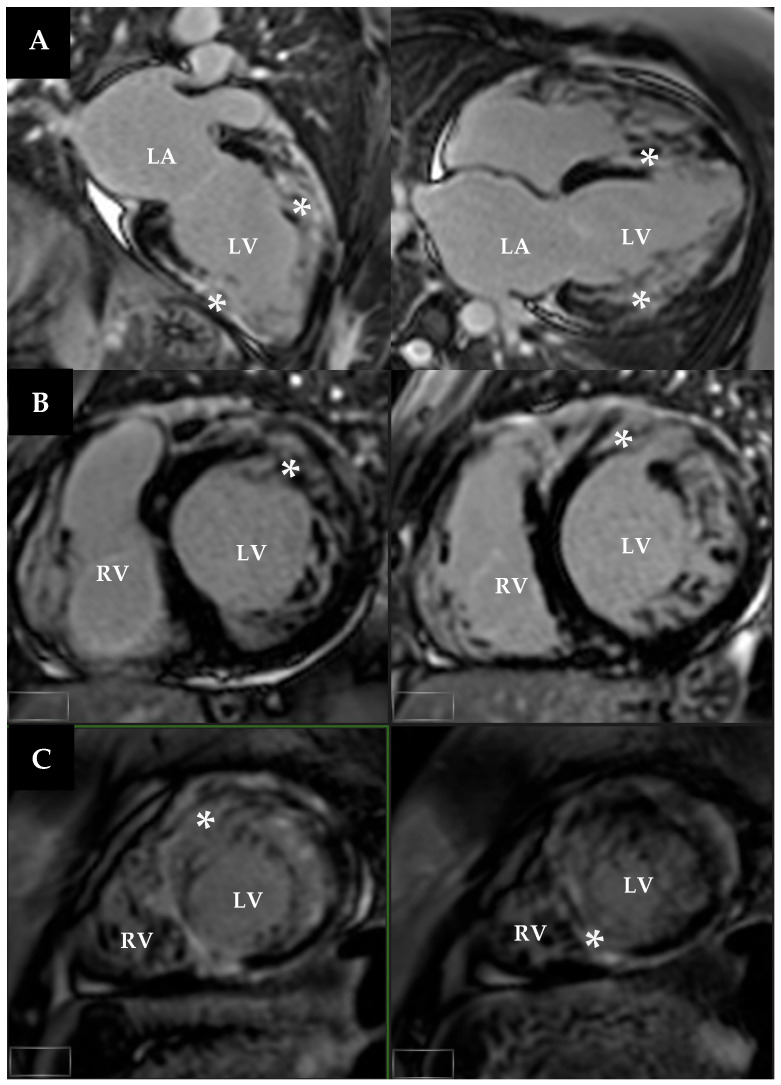
Patchy LGE distribution (asterisks), predominantly localized to the most hypertrophied myocardial regions and the RV insertion points. (**A**) Two− and 4−chamber CMR views. (**B**,**C**) Short−axis CMR views. CMR: Cardiac magnetic resonance; LGE: Late gadolinium enhancement; LV: Left ventricle; RV: Right ventricle. CMR: Cardiac magnetic resonance; LA: Left atrium; LGE: Late gadolinium enhancement; LV: Left ventricle.

In HCM, LGE is detectable in 40–80% of patients [6,56,57,58,59,159] and is more prevalent in gene-positive patients [57]. While the distribution of LGE may be variable, it characteristically exhibits a mid-wall pattern within the most hypertrophied myocardial segments and at the RV insertion regions (Figure 10) [6,56,59]. Moreover, LGE may involve cardiac structures beyond the LV, such as the RV wall, or might, more rarely, be confined to the papillary muscles [6]. No significant differences in LGE prevalence have been reported between patients with obstructive and non-obstructive HCM [160,161].

**Figure 11:**
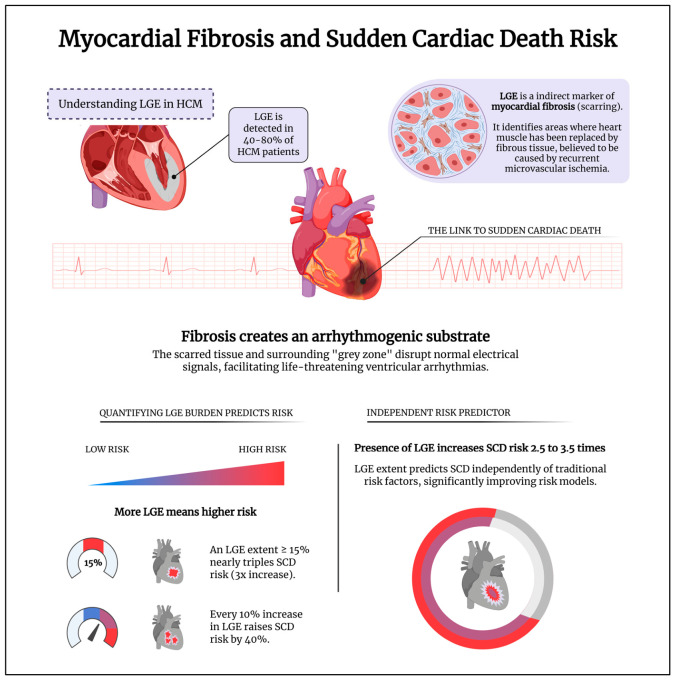
Potential prognostic impact of LGE regarding SCD. HCM: Hypertrophic cardiomyopathy; LGE: Late gadolinium enhancement; SCD: Sudden cardiac death.

LGE has been consistently associated with an increased risk of SCD (Figure 11) [57,59,60,61,62]. Early studies demonstrated markedly higher odds of cardiac death and SCD in patients with LGE [59] and subsequent large meta-analyses have confirmed this association. Across multiple pooled analyses, the presence of LGE is associated with a significantly increased risk of SCD or related arrhythmic events (OR: 2.5 to 3.5) [61,63,64,65,66]. These findings are robust and show minimal heterogeneity between studies.

Beyond its mere presence, quantitative assessment of LGE provides incremental prognostic value [67]. Several studies have demonstrated a dose–response relationship between LGE extent and arrhythmic risk. In low- and intermediate-risk patients with preserved LVEF, an LGE extent ≥ 15% of LV mass is associated with a near threefold increase in SCD or aborted SCD, irrespective of obstructive status [68]. In low- and intermediate-risk patients, even lower thresholds (>5–10% of LV mass) have been linked to a markedly elevated long-term risk of malignant ventricular arrhythmias [69]. At the same time, absent or minimal LGE has been associated with a favourable prognosis [70]. Consistent with these observations, a pooled HR of 1.56 for SCD has been reported for every 10% increase in LGE extent [64].

Significantly, LGE extent predicts SCD independently of traditional clinical risk factors. Event rates rise proportionally with increasing LGE extent, with each 10% increase approximately corresponding to a 40% relative increase in SCD risk [56]. Notably, a substantial proportion of SCD events occur in patients classified as low risk by conventional criteria, underscoring the critical role of LGE in refining SCD risk stratification in HCM [56].

There is currently no consensus on the optimal method for quantifying LGE in HCM. Among the available techniques, the 2-SD method remains the only approach validated against histopathological findings [162] and is therefore recommended in current clinical practice guidelines [1]. Nevertheless, accumulating evidence indicates that several quantitative approaches (including manual contouring and the 2-SD, 4-SD, and 6-SD techniques) exhibit comparable prognostic performance for predicting SCD [163].

Reflecting this growing body of evidence, current European and American guidelines recognize the prognostic value of LGE extent in HCM. ESC guidelines suggest considering ICD implantation when LGE involves ≥15% of LV mass [1], while ACC/AHA guidelines allow ICD consideration in patients with extensive LGE and intermediate risk [2] (Table 2). However, the 15% threshold is increasingly questioned, as recent studies show that lower LGE extents (≈5–10%) are also associated with higher SCD risk and may outperform the 15% cut-off [164], with LGE extent often providing incremental prognostic value beyond established risk models [164,165,166].

Significantly, the prognostic significance of LGE extends beyond its absolute extent to encompass its spatial distribution, morphological pattern, and internal heterogeneity. Studies characterizing LGE patterns in HCM have shown that ischemic LGE is associated with a worse prognosis than non-ischemic LGE, underscoring the heterogeneity of myocardial fibrosis and its differential arrhythmogenic potential [167]. In addition, scar heterogeneity itself appears to convey independent prognostic information. Aquaro et al. introduced a novel LGE-dispersion mapping technique that quantifies fibrosis heterogeneity by assessing pixel-level signal-intensity variability and deriving a global dispersion score (GDS). In patients with low-to-intermediate 5-year SCD risk, a GDS score > 0.86 was associated with significantly worse outcomes. This provided incremental risk stratification beyond LGE extent > 15%, yielding a meaningful net reclassification improvement [168]. More recently, detailed characterization of scar architecture using LGE-CMR post-processing has further highlighted the prognostic importance of fibrosis organization. Quantification of scar components (including dense core, border zone (BZ), and BZ channels) has been shown to provide an independent prediction of ventricular arrhythmias in HCM, significantly improving the predictive performance of both ESC and AHA/ACC SCD risk models. These findings suggest that the arrhythmogenic potential of myocardial fibrosis is determined not only by its extent but also by the complexity of its structural organization, particularly by the presence of conducting channels within heterogeneous scar tissue [169].

Finally, the absence of LGE indicates a relatively favourable prognosis. However, the risk of SCD is not completely abolished [56,59,66,170]. Minimal LGE involvement (1–5% of LV mass) does not appear to confer a significant increase in SCD risk [56,70], and isolated LGE confined to RV insertion points has not been associated with adverse arrhythmic outcomes [56].

Progression of myocardial fibrosis in HCM has been consistently associated with adverse remodelling, including deterioration of LVEF below 50% and increased rates of HF hospitalization [59,152,159,171,172,173,174]. Baseline markers of disease severity (including MWT, LV mass, and baseline LGE extent) have been identified as predictors of subsequent fibrosis progression [173]. Although genotype positivity alone does not appear to predict LGE progression, patients who are both genotype- and phenotype-positive exhibit greater fibrosis progression compared with genotype-negative individuals. Importantly, LGE progression is accompanied by adverse functional remodelling, including increases in LV end-diastolic volume and declines in LVEF and GLS [173].

Evidence regarding the impact of therapeutic interventions on myocardial fibrosis remains limited. Notably, in a subgroup of patients who underwent surgical myectomy, the LGE threshold beyond which the risk of 5-year primary events increased was substantially higher (approximately ≥25%), suggesting a potential protective effect of this SRT [68]. Emerging data on disease-modifying therapies are also encouraging. A recent randomized study reported a significant reduction in LGE extent with CMI mavacamten compared with placebo [175]. However, these findings require confirmation in larger cohorts with longer follow-up.

## 11. Mapping Sequences

Recent studies have explored the prognostic role of advanced CMR tissue characterization beyond LGE in HCM. Global native T1 and extracellular volume (ECV) quantified outside LGE regions are independently associated with HF–related events, although not with SCD [176]. While several studies suggest that ECV quantification may have potential value in predicting SCD risk, evidence remains heterogeneous [157,177,178].

**Figure 12:**
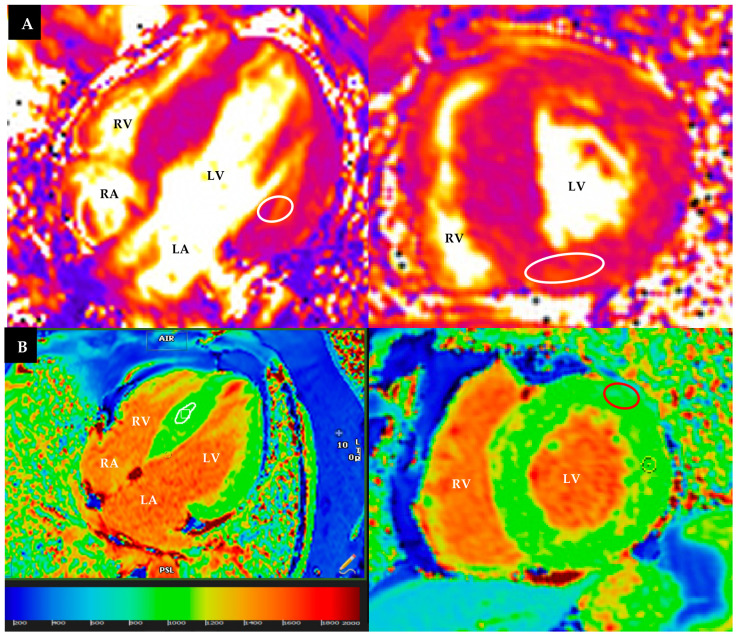
(**A**) T2 mapping sequences in 4−chamber and short−axis CMR views. The white circles indicate regions of interest with elevated T2 values. (**B**) Noncontrast native T1 mapping in 4−chamber and short−axis CMR views. The white circle indicates a region of interest with elevated T1 values (1120 ms), and the red circle indicates a region with normal T1 values (980 ms). CMR: Cardiac magnetic resonance; LA: Left atrium; LV: Left ventricle; RA: Right atrium; RV: Right ventricle.

Noncontrast native T1 mapping has also emerged as a surrogate marker of diffuse myocardial fibrosis (Figure 12) [179,180], with significantly elevated values observed in HCM patients compared with controls, even in myocardial segments without LGE [179]. In addition, T2-weighted imaging may provide complementary risk information (Figure 12). In a multicentre study, patients exhibiting increased signal intensity on T2-weighted sequences were more frequently classified as intermediate-to-high risk according to ESC and ACC/AHA SCD algorithms and showed higher projected SCD rates than those without T2 abnormalities [181]. However, further large-scale and longitudinal studies are required, as evidence regarding ECV and mapping techniques remains promising but insufficient to guide clinical decision-making at present.

## 12. Microcirculation

It is well established that patients with HCM exhibit microvascular dysfunction, resulting in impaired myocardial blood flow (MBF), which in turn contributes to ischemia-mediated myocyte death and replacement fibrosis. Ischemia can be associated with LV adverse remodelling, systolic and diastolic dysfunction, and their complications, including the potential contribution to SCD [182,183], as lower MBF has been proven to be an independent predictor of cardiovascular mortality [184]. Ischemia assessment is primarily performed using positron emission tomography (PET) and quantitative perfusion CMR imaging. In PET imaging, the ratio of basal MBF to MBF during pharmacological vasodilation allows calculation of coronary flow reserve. In HCM, resting MBF is similar to that of healthy individuals, but the increase in blood flow after pharmacological vasodilation is significantly impaired. On the other hand, perfusion CMR provides superior spatial resolution and also demonstrates blunted stress MBF in patients with HCM [183]. With both imaging techniques, MBF impairment can affect both hypertrophied and non-hypertrophied segments and is more pronounced in the subendocardium [183,185].

Using quantitative perfusion CMR imaging, fibrotic areas (assessed by LGE) exhibit reduced MBF [184]. HCM patients, including 20% of genotype-positive/phenotype-negative individuals [186], exhibit significantly lower stress myocardial blood flow and myocardial perfusion reserve (both *p* < 0.001) than healthy controls [187].

Altogether, PET and CMR imaging may provide complementary mechanistic information on microcirculatory impairment. However, the contribution of ischemia to the pathogenesis of malignant arrhythmias remains incompletely elucidated.

## 13. Pulmonary Hypertension

Pulmonary hypertension (PH) is a common complication in HCM, with a prevalence of about 12–38% [188,189], and is linked to poorer functional status and adverse outcomes, including higher mortality [190].

From an echocardiographic standpoint, the coexistence of PH and HCM has been associated with a prolonged resting E-wave DT and a higher prevalence of significant MR. These findings reflect the underlying pathophysiology of PH in HCM as a manifestation of left-sided heart disease, which is driven primarily by LV diastolic dysfunction and chronically elevated LA pressures [189,191].

PH in HCM is associated with adverse clinical outcomes, including poorer functional status, higher rates of AF, thromboembolism, HF, and biventricular dysfunction [188,192]. Although direct evidence linking PH to arrhythmic events is limited, exercise-induced PH has been associated with MACE, including HF, AF, and sustained VT [191].

Overall, PH confers a worse prognosis and increased mortality in HCM [190].

## 14. Stress Echocardiography

Stress exercise echocardiography (SEE) evaluates cardiac anatomy and haemodynamics during dynamic, physiological exertion using ultrasound imaging. In HCM, it is particularly valuable for unmasking exercise-induced LVOTO [193] and guiding clinical management. In contrast, pharmacological provocation with dobutamine is not recommended because it does not accurately reflect physiological conditions [1,2].

According to current guidelines, SEE carries a Class I indication for the assessment of myocardial ischemia and LVOTO when bedside manoeuvres fail to generate a gradient > 50 mmHg, as well as for evaluating SAM of the mitral valve in symptomatic patients [1,2].

There is also evidence supporting its key role in assessing functional capacity and risk stratification in asymptomatic patients. Reduced exercise capacity and abnormal heart rate recovery predict adverse outcomes in HCM, whereas echocardiographic parameters do not [194]. Evidence supporting the use of SEE for arrhythmic risk or SCD prediction remains limited.

## 15. Future Directions

In the era of artificial intelligence (AI), new data have emerged regarding its potential use for diagnostic and risk-stratification purposes in HCM. Within this field, Fahmy et al. developed an AI model combining radiomics and deep learning, using balanced steady-state free precession cine images, that was able to detect myocardial scar in patients with HCM before gadolinium administration, thereby potentially decreasing the need for contrast administration [195]. A few years later, the same group demonstrated that LV radiomic features derived from LGE imaging are significantly associated with SCD risk and offer incremental prognostic value beyond current guideline-based risk models [196].

CMR diffusion tensor imaging has emerged as a promising in vivo technique for detecting myocardial disarray and microstructural abnormalities in HCM by quantifying mean diffusivity (MD), fractional anisotropy (FA), and second eigenvector angle, even in the absence of hypertrophy or fibrosis [197,198,199,200]. Compared with healthy controls, HCM patients exhibit lower FA, higher MD, and increased sheetlet angulation, reflecting greater myocyte disarray and altered myocardial architecture [187]. Notably, impaired FA has been associated with an increased risk of ventricular arrhythmias [201], and similar (though less pronounced) microstructural abnormalities have been identified in genotype-positive/phenotype-negative individuals [187]. While these findings are encouraging, further studies are needed to establish the clinical utility and prognostic value of diffusion tensor imaging in HCM.

## 16. Conclusions

HCM is the most common genetic cardiomyopathy and a leading cause of SCD in young adults. Although significant progress has been made in elucidating its pathophysiology and refining diagnostic and risk-prediction tools, accurate stratification of SCD risk remains challenging. Importantly, there is still no global consensus on the primary prevention of ICD implantation. Current guidelines diverge in the weight they assign to traditional risk markers and have yet to integrate several emerging imaging-based predictors supported by recent evidence. Furthermore, uncertainties persist regarding the prognostic and arrhythmic effects of novel CMIs.

**Figure 13:**
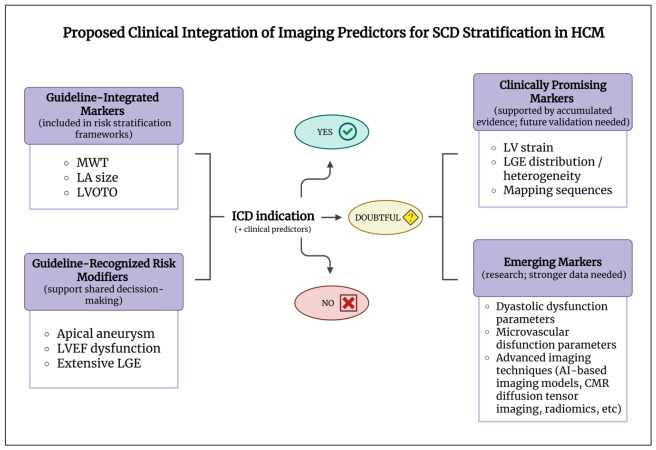
Proposed clinical integration of imaging predictors for SCD stratification in HCM. AI: Artificial intelligence; CMR: Cardiac magnetic resonance; HCM: Hypertrophic cardiomyopathy; ICD: Implantable cardioverter-defibrillator; LA: Left atrium; LGE: Late gadolinium enhancement; LV: Left ventricle; LVEF: Left ventricular ejection fraction; LVOTO: Left ventricular outflow tract obstruction; MWT: Myocardial wall thickness; SCD: Sudden cardiac death.

This narrative review synthesizes current evidence on the principal imaging-derived risk markers for SCD in HCM (Figure 13), to inform future research and advance toward more individualized, precise, and evidence-based approaches to SCD risk stratification.

## Figures and Tables

**Table 1:** Main predictors of sudden cardiac death risk.

Predictor	Pathophysiology	Adverse Events/Associations	Key Messages	References
MWT	Greater myocardial disorganization, ischemia	NSVT, SCD, LVOTO, HF progression	Maximal MWT is a continuous, not dichotomous, risk marker; insufficient alone for SCD stratification	[11,12,13,14,15,16,17,18,19]
LVOTO	Dynamic obstruction from septal hypertrophy and SAM: increased wall stress, ischemia, and elevated filling pressures	SCD, ventricular arrhythmias, HF progression, AF	Primarily a functional rather than an arrhythmic predictor	[11,15,18,20,21,22,23,24]
Apical aneurysm	Fibrous border + intraventricular gradient	SCD, VT, appropriated ICD discharge, HF, MACE	ACC/AHA: independent predictor; ESC: not sufficiently strong as an isolated predictor	[25,26,27,28,29,30,31,32]
LA size	SAM/MR→atrial myopathy	AF, MACE	LA size and function reflect disease severity; modest direct SCD signal, strong AF/HF marker	[33,34,35,36,37,38,39,40]
Systolic LV dysfunction	End-stage remodelling with wall thinning and fibrosis	SCD, malignant arrhythmias, advanced HF, death	ACC/AHA: ICD if <50% + risk factors (class IIa); not included in ESC	[41,42,43]
Diastolic LV dysfunction	Increased LV filling pressure	SCD, AF, HF, MACE	Robust prognostic marker; indirect but relevant contributor to arrhythmic risk	[44,45,46,47,48,49]
GLS	Subclinical dysfunction, fibrosis	VT, SCD, appropriate ICD discharge, HF, MACE	Predictive even in patients with an ESC risk score < 6%	[38,50,51,52,53,54,55]
LGE	Replacement and interstitial fibrosis from ischemia and myocyte loss	VT, SCD, appropriate ICD discharge	LGE absence: low SCD risk but not zero. Better predictor than ESC/ACC in many studies	[6,56,57,58,59,60,61,62,63,64,65,66,67,68,69,70]

ACC: American College of Cardiology; AF: atrial fibrillation; AHA: American Heart Association; ESC: European Society of Cardiology; GLS: Global longitudinal strain; HF: Heart failure; ICD: Implantable cardiac defibrillator; LA: Left atrium; LGE: Late gadolinium enhancement; LV: Left ventricle; LVOTO: Left ventricle outflow tract obstruction; MACE: Major adverse cardiovascular event; MR: Mitral regurgitation; MWT: Myocardial wall thickness; NSVT: Non sustained ventricular tachycardia; SAM: Systolic anterior movement; SCD: Sudden cardiac death; VT: Ventricular tachycardia.

**Table 2:** Guideline-supported imaging predictors of SCD: Comparison between ESC and ACC/AHA guidelines.

Predictor	ESC	ACC/AHA	Interpretation/Key Differences
MWT	Yes	Yes	Greater MWT is linked to higher risk. ACC/AHA: MWT ≥ 30 mm (massive LV hypertrophy) qualifies as Class IIa ICD indication, even in isolation.
LVOTO	Yes	No	ESC: Greater LVOT is linked to higher risk.
Apical aneurysm	No	Yes	ACC/AHA: Class IIa ICD indication, even in isolation.
LA size	Yes	Yes	Greater LA size (diameter) is linked to higher SCD risk.
Systolic LV dysfunction (EF < 50%)	Considered	Yes	ESC: Additional risk factor (not included in the calculator); Class IIb ICD indication for selected low-risk patients. ACC/AHA: Class IIa ICD indication, even in isolation
Extensive LGE (≥15%)	Considered	Yes	ESC: Class IIb ICD indication for selected low-risk patients. ACC/AHA: Class IIa ICD indication, even in isolation

ACC: American College of Cardiology; AHA: American Heart Association; EF: Ejection fraction; ESC: European Society of Cardiology; GLS: Global longitudinal strain; ICD: Implantable cardioverter-defibrillator; LA: Left atrium; LGE: Late gadolinium enhancement; LV: Left ventricle; LVOTO: Left ventricle outflow tract obstruction; MWT: Myocardial wall thickness; SCD: Sudden cardiac death.

## Data Availability

No new data were created or analysed in this study. Data sharing is not applicable to this article.

## Abbreviations

The following abbreviations are used in this manuscript:

ACC	American College of Cardiology
AHA	American Heart Association
AF	Atrial fibrillation
AI	Artificial intelligence
ASA	Alcohol septal ablation
BZ	Border zone
CMI	Cardiac myosin inhibitor
CMR	Cardiac magnetic resonance
CMR-FT	Cardiac magnetic resonance feature tracking
DT	Deceleration time
ECV	Extracellular volume
ESC	European Society of Cardiology
FA	Fractional anisotropy
GCS	Global circumferential strain
GDS	Global Dispersion Score
GLS	Global longitudinal strain
GRS	Global radial strain
HCM	Hypertrophic cardiomyopathy
HF	Heart failure
HR	Hazard ratio
ICD	Implantable cardioverter-defibrillator
LA	Left atrium
LACS	Left atrial conduit strain
LARS	Left atrial reservoir strain
LAS	Left atrial strain
LASb	Left atrial strain booster
LASc	Left atrial strain conduit
LASr	Left atrial strain reservoir
LAVI	Left atrial volume index
LGE	Late gadolinium enhancement
LV	Left ventricle/Left ventricular
LVEF	Left ventricular ejection fraction
LVH	Left ventricular hypertrophy
LVOT	Left ventricular outflow tract
LVOTO	Left ventricular outflow tract obstruction
MACE	Major adverse cardiovascular events
MBF	Myocardial blood flow
MD	Mean diffusivity
MR	Mitral regurgitation
MWT	Myocardial wall thickness
NSVT	Odds ratio
OR	Positron emission tomography
PET	Pulmonary hypertension
PH	Right ventricle/Right ventricular
RV	Systolic anterior motion
SAM	Sudden cardiac death
SCD	Standard deviation
SD	Stress exercise echocardiography
SEE	Septal reduction therapy
SRT	Transthoracic echocardiography
TTE	Ventricular tachycardia
VT	Left atrial strain booster
